# Novel FHL1 mutation variant identified in a patient with nonobstructive hypertrophic cardiomyopathy and myopathy – a case report

**DOI:** 10.1186/s12881-020-01131-w

**Published:** 2020-09-29

**Authors:** Adrian Giucă, Cristina Mitu, Bogdan Ovidiu Popescu, Alexandra Eugenia Bastian, Răzvan Capşa, Adriana Mursă, Viorica Rădoi, Bogdan Alexandru Popescu, Ruxandra Jurcuţ

**Affiliations:** 1 Expert Center for Rare Cardiovascular Genetic Diseases, “Prof. Dr. C.C. Iliescu” Emergency Institute for Cardiovascular Diseases, Street no.258, postal code:022328 Bucharest, Romania; 2 Neurology Department, Colentina Clinical Hospital, Bucharest, Romania; 3 “Carol Davila” University of Medicine and Pharmacy, Euroecolab, Bucharest, Romania; 4 Pathology Department, Colentina Clinical Hospital, Bucharest, Romania; 5 Fundeni Clinical Institute, Bucharest, Romania

**Keywords:** FHL1, Hypertrophic cardiomyopathy, Emery-Dreifuss muscular dystrophy, Case report

## Abstract

**Background:**

Hypertrophic cardiomyopathy (HCM) is a genetic disorder mostly caused by sarcomeric gene mutations, but almost 10% of cases are attributed to inherited metabolic and neuromuscular disorders. First described in 2008 in an American-Italian family with scapuloperoneal myopathy, FHL1 gene encodes four-and-a-half LIM domains 1 proteins which are involved in sarcomere formation, assembly and biomechanical stress sensing both in cardiac and skeletal muscle, and its mutations are responsible for a large spectrum of neuromuscular disorders (mostly myopathies) and cardiac disease, represented by HCM, either isolated, or in conjunction with neurologic and skeletal muscle impairment. We thereby report a novel mutation variant in FHL1 structure, associated with HCM and type 6 Emery-Dreifuss muscular dystrophy (EDMD).

**Case presentation:**

We describe the case of a 40 year old male patient, who was referred to our department for evaluation in the setting of NYHA II heart failure symptoms and was found to have HCM. The elevated muscular enzymes raised the suspicion of a neuromuscular disease. Rigid low spine and wasting of deltoidus, supraspinatus, infraspinatus and calf muscles were described by the neurological examination. Electromyography and muscle biopsy found evidence of chronic myopathy. Diagnosis work-up was completed by next-generation sequencing genetic testing which found a likely pathogenic mutation in the FHL1 gene (c.157-1G > A, hemizygous) involved in the development of X-linked EDMD type 6.

**Conclusion:**

This case report highlights the importance of multimodality diagnostic approach in a patient with a neuromuscular disorder and associated hypertrophic cardiomyopathy by identifying a novel mutation variant in FHL1 gene. Raising awareness of non-sarcomeric gene mutations which can lead to HCM is fundamental, because of diagnostic and clinical risk stratification challenges.

## Background

Hypertrophic cardiomyopathy (HCM) is an umbrella diagnosis which can encompass various etiologies for which the specific diagnosis can influence both the diagnostic workup and the therapeutic choices [1]. Only up to 60% of HCM patients have sarcomeric gene mutations, and for as many as 25–30% of cases the genetic etiology is not found. Mutations in the structure of four-and-a-half LIM domains 1 (FHL1) gene have been described in the last decade to be associated with a large spectrum of diseases, including Emery-Dreifuss muscular dystrophy (EDMD); X-linked scapuloperoneal myopathy (X-SPM); reducing bodies myopathies (RBM); X-linked myopathy with postural muscle atrophy (X-MPMA); rigid spine syndrome and more rarely HCM [2]. The present case report is the first description of a novel FHL1 mutation leading to a cardio-muscular phenotype.

## Case presentation

We present the case of a 40 year old Caucasian male who was referred to our department for evaluation of NYHA II heart failure symptoms and a recent persistent episode of atrial fibrillation (AF). Clinical examination shows irregular heart rate and mild pansystolic apical murmur.

**Fig. 1:**
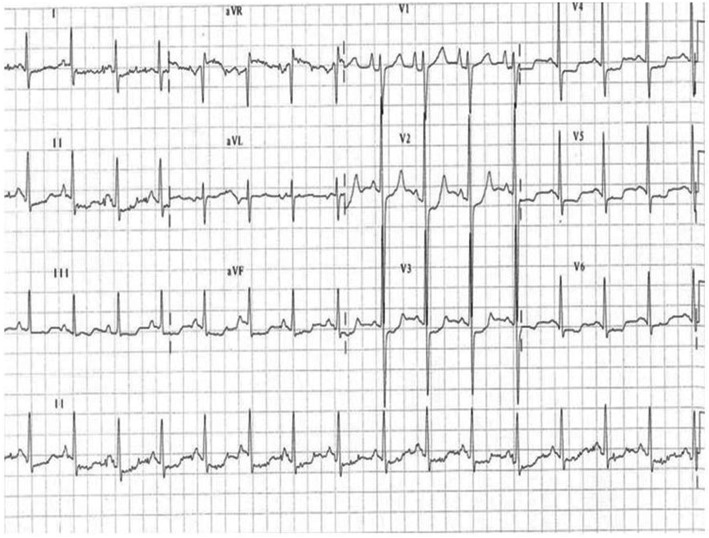
Electrocardiogram shows sinus tachycardia, enlarged right atrium with left ventricular hypertrophy and secondary repolarization abnormalities

Laboratory tests identified high brain natriuretic peptide levels (BNP) (784 pg/ml) and slightly elevated muscular enzymes (CK 227.3 U/l). Serial electrocardiographic (ECG) tracings indicate both sinus rhythm and AF episodes, with left ventricular (LV) hypertrophy voltage criteria (Fig. 1).

**Fig. 2:**
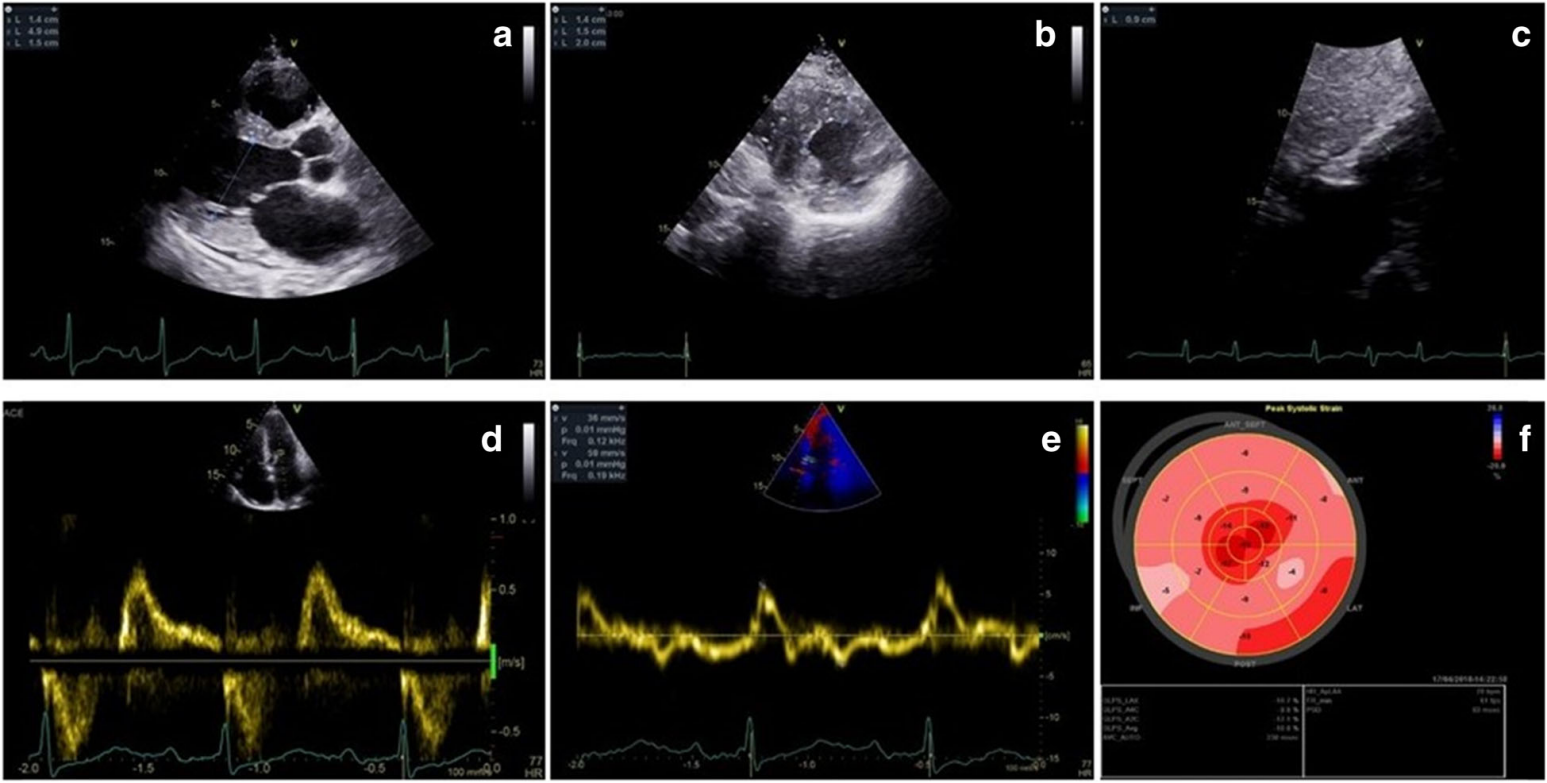
Transthoracic 2D echocardiography. **a.** Parasternal long-axis section revealing concentric LV hypertrophy. **b.** Parasternal midventricular short axis with maximal wall thickness of 20 mm at inferoseptal level. **c.** Subcostal view with RV free wall hypertrophy (9 mm). **d.** Transmitral flow Doppler interrogation finds grade 3 diastolic dysfunction. **e.** Myocardial Doppler interrogation of the septal mitral annulus shows low longitudinal systolic and diastolic velocities. **f.** Bull’s eye LV myocardial deformation map with low global longitudinal strain and apical sparring

**Fig. 3:**
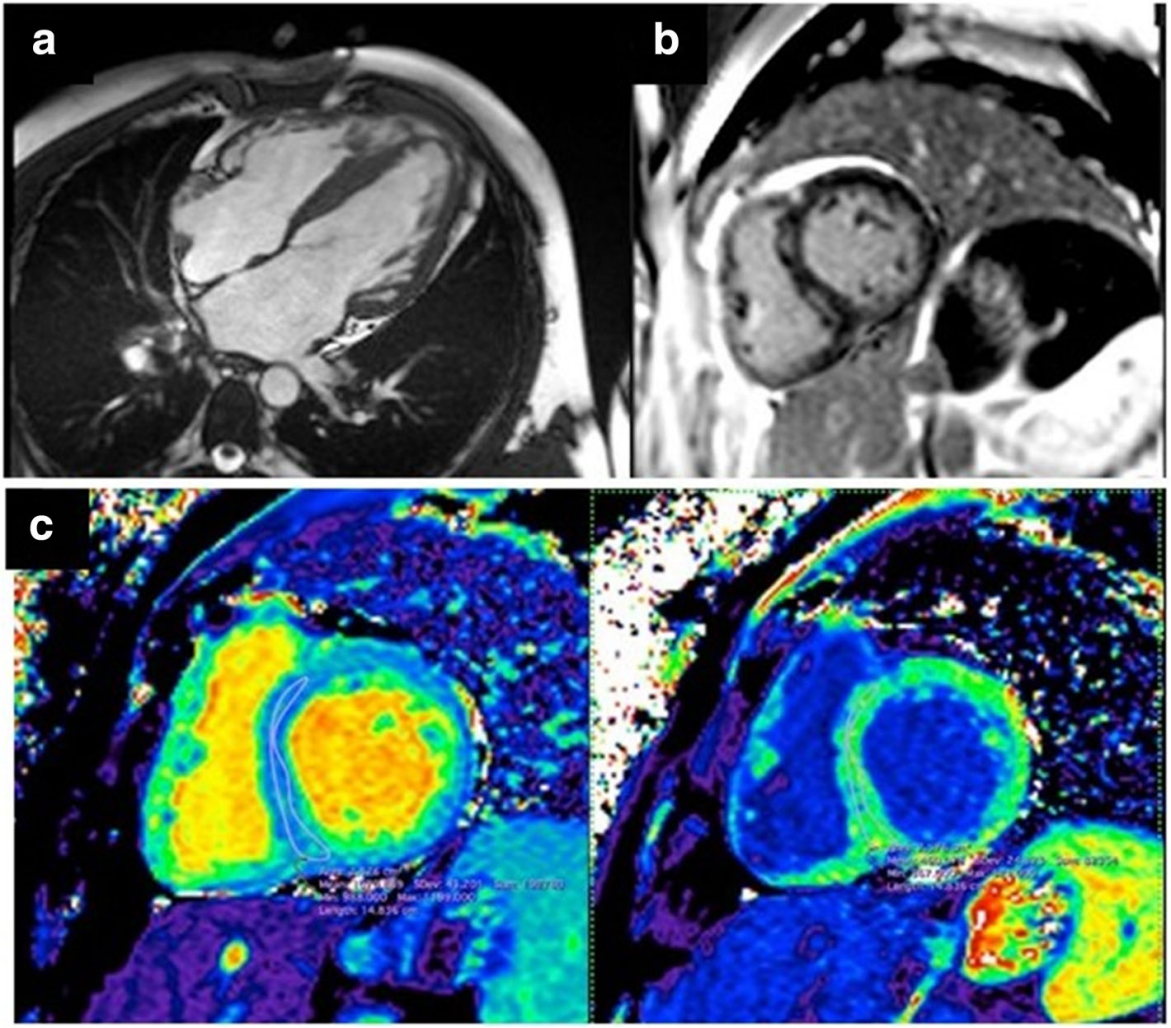
Cardiac MRI. **a**. Biventricular hypertrophy with maximal wall thickness of 19 mm at inferoseptal level; **b**. Late gadolinium enhancement basal mid-myocardial disposition. C. Diffuse high T1 values (1074 ms precontrast administration) with extracellular volume expansion to 37%

Transthoracic echocardiography (TTE) (Fig. 2) revealed moderate biventricular thickening (maximal LV wall thickness 20 mm at inferoseptal level, 15 mm in most other segments with symmetrical disposition, right ventricular (RV) free wall 9 mm), biatrial dilatation, important longitudinal dysfunction with apical sparring, preserved LV ejection fraction (LVEF), but severe impairment of diastolic function with restrictive physiology. Cardiac magnetic resonance (CMR) confirmed biventricular hypertrophy with maximal wall thickness of 19 mm at inferoseptal level and found late gadolinium enhancement (LGE) with basal mid-myocardial disposition. It also described diffuse high T1 values (1074 ms precontrast administration) with extracellular volume expansion to 37% (Fig. 3). Systemic amyloidosis was excluded by negative whole-body Tc^99m^ Hydroxymethylene Diphosphonate (99Tc-HMDP) scintigraphy, absence of monoclonal gammapathy and multiple tissue biopsies.

**Fig. 4:**
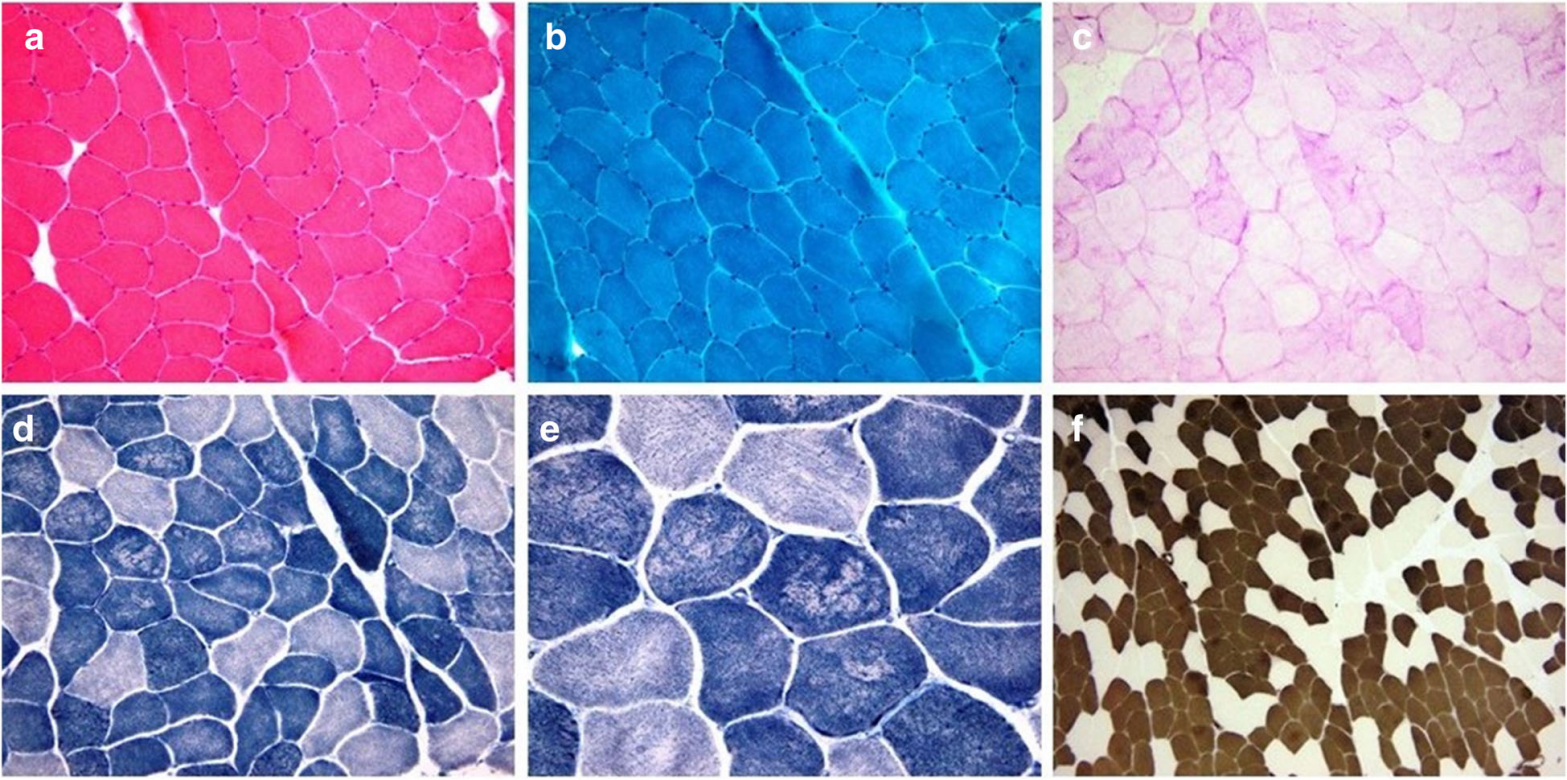
Cryosections of the left deltoid muscle biopsy specimen, frozen in isopentane cooled in liquid nitrogen were used for routine histological staining and histochemical techniques. Hematoxylin and Eosin (HE 200x, panel **a**), Gomori trichrome (GT 200x, panel **b**) and Periodic Acid Schiff (PAS 200x, panel **c**) staining revealed unspecific minimal myopathic changes with mild variation in the fiber size and rare atrophic, angulated fibers. On reduced nicotinamide adenine dinucleotide tetrazolium reductase (NADH-TR 200x, panel D and 400x, panel **e**) unevenness of stain and multiple core-like areas devoid of enzyme activity in both fiber types, but mainly in type I fibers were identified. A nonspecific predominance of type I fibers (dark color) was highlighted by adenosine triphosphatase staining (ATPase pH 4,35,100x, panel **f**)

Due to the constant increase in CK (in the absence of muscular symptoms), a neurologic examination was performed and revealed decreased facial expression, slight dysphonia, mild weakness involving shoulder and pelvic girdles and also peroneal muscles. We noticed slight wasting of deltoidus, supraspinatus, infraspinatus and calf muscles, diminished deep tendon reflexes and reduced mobility of the lumbar spine (rigid low spine) with no others joint contractures. Electromyography was performed with evidence of chronic myopathy. Left deltoid muscle biopsy showed non-specific myopatic findings, but, also, on reduced nicotinamide adenine dinucleotide tetrazolium reductase (NADH-TR) and other oxidative enzyme stainings, unevenness of stain and multiple core-like areas devoid of enzyme activity in both fiber types, mainly in type I fibers, morphological signs of abnormal internal architecture with sarcomeric disorganization (Fig. 4). No signs of necrosis, regeneration, mitochondrial abnormalities, fibrosis or amyloid deposits were noticed; no rimmed vacuoles or reducing bodies were identified. The diagnostic workup was completed by genetic testing by next-generation sequencing with a comprehensive cardiomyopathy panel, which found a likely pathogenic mutation in the FHL1 gene (c.157-1G > A, hemizygous, using the NM_001449.4 transcript) involved in the development of X-linked EDMD type 6. The mutation classification was based on the fact that this sequence change affects an acceptor splice site in intron 3 of the FHL1 gene, and therefore is expected to disrupt RNA splicing and likely results in an absent or disrupted protein product.

**Fig. 5:**
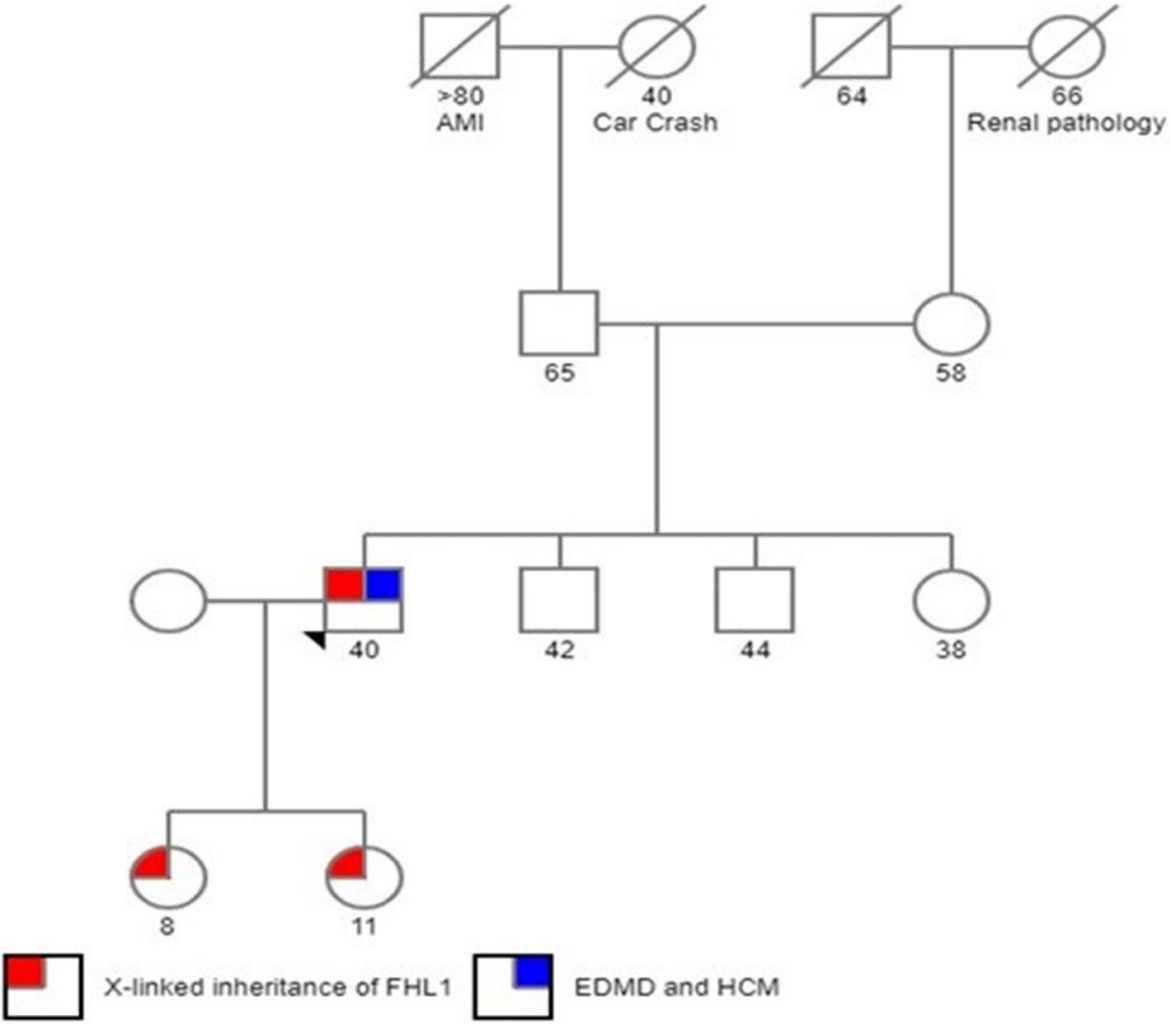
Pedigree of the family with X-linked transmission

Analyzing the pedigree of the index case, we evaluated the 2 daughters who are obligate carriers (clinical examination, ECG, TTE) and found no cardiac or muscular abnormalities (Fig. 5). The patient’s mother was hypertensive but no specific electrocardiographic, echocardiographic and neurological changes were found and she refused further testing, including genetic testing.

## Discussion and conclusions

The present case highlights the complex differential diagnosis of biventricular hypertrophy in a young man, as well as the importance of extracardiac red flags in reaching the specific etiology. While longitudinal dysfunction and apical sparring, together with the neurologic manifestations, first prompted searching for systemic amyloidosis as a phenocopy, the non-specific pattern of LGE and the negative scintigraphy and tissue biopsies, made it an unlikely occurrence. The elevated muscular enzymes alongside mild skeletal-muscle function alteration raised the suspicion of a neuromuscular disease as the cardiomyopathy’s etiology, with further genetic confirmation.

Synthetized by a gene located on Xq26.3, FHL proteins are composed of a variable number of LIM domains encoded by 8 exons (alternate splicing of the last 6 leads to 3 different proteic isoforms) [3, 4]. FHL1 is the most important and plays a fundamental role in the synthesis and assembly of the sarcomere, in biomechanical stress sensing and binds to different ultracellular structures [3].

Although associated with a large number of diseases, FHL1 mutations can be divided into 2 large groups depending on the presence or absence of reducing-bodies (RBs) on muscle biopsy specimens [2]. The site of mutation is very important, as anomalies located in the distal exons, which are responsible for the synthesis of the third and fourth LIM domains, lead to non-RBs disorders, like HCM and EDMD [2, 5].

EDMD is a rare disease, manifesting by the following triad: early joint contractures of Achiles tendons, elbows and rigid spine; childhood onset of muscle weakness and wasting; and adult-onset cardiomyopathy. It is inherited either in a X-linked pattern (following mutations in FHL1 or EMD), or an autosomal dominant pattern (caused by alterations in the structure of lamin A/C (LMNA)) [4]. In our case, we discovered a novel mutation variant in the FHL1 gene (c.157-1G > A, splice acceptor, hemizygous) not previously reported in the literature, which led to nonobstructive HCM and skeletal myopathy. This patient’s variant affects an acceptor splice site in intron 3 of the FHL1 gene; it is expected to disrupt RNA splicing and likely results in an abnormal protein product. Although EDMD patients may exhibit various phenotypes, it is important to note that our case shows neither neck rigidity nor limb joints involvement.

The most frequent gene mutations which cause EDMD remain emerin and lamin A/C, and they evolve with dilated cardiomyopathy. Geuneau et al. were the first to report on the involvement of FHL1 mutations in EDMD in 2009 [4]. After analyzing 6 families with FHL1 related EDMD, the authors found that all index cases had cardiac involvement (manifested by arrhythmia, hypertrophy and/or conduction disorders), concluding that HCM phenotype in patients with muscular disease should prompt for targeted screening for FHL1 [4]. Most heterozygous female carriers were either asymptomatic or had mild cardiac/muscular impairment [4]. A few years later, the first report of the association of a predominantly distal myopathy with HCM occurring secondary to an FHL1 mutation further expanded the clinical spectrum of FHL1-related myopathies [6].

Conversely, after testing 121 HCM patients without known sarcomeric mutations, Friedreich et al. provided evidence for FHL1 mutations as causative for HCM with or without associated myopathy in 7 unrelated families and proposed it as a novel disease gene to be searched in isolated HCM without myopathy [3]. Binder et al. also reported on 12 male patients with an FHL1 mutation related specific form of cardiomyopathy with midventricular and apical hypertrophy, fibrosis, and a spongious structure [7]. Of note, mild persistent elevation of plasmatic CK was an universal finding in all adult males and in some of the related females in a large reported family of FHL1 mutation with associated cardiomyopathy as first presentation [8]. Interestingly, in our patient’s case, the muscular changes became clinically relevant 1 year after the cardiac presentation, but mild CK elevation was the first red flag for etiologic diagnosis.

Risk stratification of sudden cardiac death in HCM is based on a risk score calculator which does not include genetic mutations as a possible determinant [1]. The specific risk profile of FHL1 related HCM is less studied; however, several groups reported on sudden cardiac death (SCD) as an early complication in these patients, even during childhood [8–10]. However, all published data come from case reports and case series, and cannot lead to a change in current intracardiac defibrillator (ICD) implantation guidelines in primary prevention of SCD. This underlines the importance of registries in rare diseases for describing the clinical characteristics and the long-term outcome of these patients.

The present case report highlights the fact that clinicians should be aware of more than the common sarcomeric gene mutations causing HCM. Focusing on red-flags during the diagnostic workup (e.g. increased muscular enzymes, subclinical skeletal muscular dysfunction), mutations in other genes related to muscular diseases, as FHL1, are a possible cause. More efforts should be put in developing registries for rare cardiomyopathies in order to understand the associated risk profile.

## Data Availability

The genetic laboratory where the genetic test was performed shares all data with ClinVar and this specific variant was submitted here: https://www.ncbi.nlm.nih.gov/clinvar/variation/967181/. In addition, the GenBank reference for that transcript can be found here: https://www.ncbi.nlm.nih.gov/nuccore/NM_001449.4.

## Abbreviations

HCM: Hypertrophic cardiomyopathy
FHL1: Four-and-a-half LIM domains 1
EDMD: Emery-Dreifuss muscular dystrophy
NYHA: New York Heart Association
X-SPM: X-linked scapuloperoneal myopathy
RBM: Reducing bodies myopathies
X-MBMA: X-linked myopathy with postural muscle atrophy
AF: Atrial fibrillation
BNP: Brain natriuretic peptide
CK: Creatine-kinase
ECG: Electrocardiogram
LV: Left ventricle
TTE: Transthoracic echocardiography
RV: Right ventricle
LVEF: Left ventricle ejection fraction
CMR: Cardiac magnetic resonance
LGE: Late-Gadolinium enhancement
99Tc-HMDP: Tc-99 m Hydroxymethylene Diphosphonate
RBs: Reducing bodies
LMNA: Laminin A/C
RNA: Ribonucleic acid
SCD: Sudden cardiac death
ICD: Intracardiac defibrillator
